# Automatic Identification of Systolic Time Intervals in Seismocardiogram

**DOI:** 10.1038/srep37524

**Published:** 2016-11-22

**Authors:** Ghufran Shafiq, Sivanagaraja Tatinati, Wei Tech Ang, Kalyana C. Veluvolu

**Affiliations:** 1 School of Electronics Engineering, Kyungpook National University, Daegu, 702-701, South Korea; 2 School of Mechanical and Aerospace Engineering, Nanyang Technological University, 639798, Singapore

## Abstract

Continuous and non-invasive monitoring of hemodynamic parameters through unobtrusive wearable sensors can potentially aid in early detection of cardiac abnormalities, and provides a viable solution for long-term follow-up of patients with chronic cardiovascular diseases without disrupting the daily life activities. Electrocardiogram (ECG) and siesmocardiogram (SCG) signals can be readily acquired from light-weight electrodes and accelerometers respectively, which can be employed to derive systolic time intervals (STI). For this purpose, automated and accurate annotation of the relevant peaks in these signals is required, which is challenging due to the inter-subject morphological variability and noise prone nature of SCG signal. In this paper, an approach is proposed to automatically annotate the desired peaks in SCG signal that are related to STI by utilizing the information of peak detected in the sliding template to narrow-down the search for the desired peak in actual SCG signal. Experimental validation of this approach performed in conventional/controlled supine and realistic/challenging seated conditions, containing over 5600 heart beat cycles shows good performance and robustness of the proposed approach in noisy conditions. Automated measurement of STI in wearable configuration can provide a quantified cardiac health index for long-term monitoring of patients, elderly people at risk and health-enthusiasts.

Cardiovascular diseases (CVD) are the leading cause of deaths worldwide; while nearly half of the sudden cardiac deaths occur even before medical attention is received^1^,^2^. However, effective treatment becomes possible only if timely detection is made. In this regard, continuous monitoring of hemodynamic parameters in wearable configuration serves as a viable solution. Non-invasive and unobtrusive monitoring of such parameters by wearable sensors can timely detect the onset of CVD as well as improve the quality of life for the CVD patients without disrupting their daily life activities. One set of such hemodynamic parameters is Systolic Time Interval (STI) that can indicate the underlying cardiac condition even before the appearance of physical symptoms. STI can be derived from Electrocardiogram (ECG) and Seismocardiogram (SCG) signals that can be recorded in wearable configuration^3^,^4^,^5^,^6^.

SCG is the recording of the micro-scale precordial vibrations resulting from beating heart and blood flow into the vascular tree^5^,^7^,^8^,^9^. These vibrations can be acquired by small and cost-effective accelerometers, which makes acquisition of SCG in wearable configuration feasible^3^,^4^,^5^. Further, the accelerometer employed to sense SCG can simultaneously be used in other applications such as long-term monitoring for chronic obstructive pulmonary disease (COPD) patients^10^, classification of breath disorders^11^, gait assessment for Parkinson’s disease patients^12^ and fall detection^13^,^14^ etc. SCG finds its applications in monitoring left ventricular function during ischemia^15^, magnetic field compatible alternative to ECG for cardiac stress monitoring^16^, Diagnosis of Ischemia in Patients^17^,^18^, detection of early-stage hemorrhage^19^ and atrial flutter^20^ etc.

A typical SCG signal along with the corresponding ECG signal for two consecutive beats is shown in Fig. 1(a) and ensemble averaged SCG and ECG are shown in Fig. 1(b). The peaks in the SCG signal correspond to opening and closing of aortic (AO/AC) and mitral (MO/MC) valve^21^,^22^, while the IM point occurs during the period of rapid change in ventricular pressure^23^. The intervals PEP, LVET and QS2 illustrated in Fig. 1(b) are pre-ejection period, left ventricular ejection time and electro-mechanical systole. These intervals are known as STI^24^,^25^ and its derivation from SCG and ECG has been successfully demonstrated^5^,^26^,^27^,^28^. Accurate measurement of STI is important as any deviation of STI trend can correspond to an abnormality of the heart function^25^. For instance, increased PEP and decreased LVET are shown in heart failure patients as compared to the normal subjects^29^. STI has been useful in numerous applications such as cardiac computed tomographic gating^30^, identification of exercise capacity^31^, optimization of cardiac resynchronization therapy in heart failure patients^24^, identifying extent of left ventricular (LV) muscle dysfunction^24^, observing changes in LV performance during haemodialysis^32^, mitral valve stenosis^33^, atrial fibrillation^34^, coronary artery disease^35^ and detection of ischemia^17^,^36^ etc. Further, in the context of personal health monitoring from wearable sensors, non-invasive and unobtrusive measurement of STI can provide a quantified measure of cardiac health^24^,^28^. However, deriving STI from SCG and ECG requires careful annotation for their utilization in such applications.

The annotation of peaks in SCG signal is challenging as compared to ECG due to the existence of large morphological variability of SCG among the subjects^9^ and its susceptibility to distortions from subject’s motion, respiration and noise artifacts. Since annotation of ECG signal is well established, the scope of this paper is limited to the annotation of SCG waveform. Previously, the researchers have annotated the SCG signals, but are limited to manual or semi-automated approaches^5^,^27^ that require human intervention and is generally quite time-consuming.

In this paper, we propose a scheme that automatically identifies the location of desired SCG peaks that are required to calculate STI. The approach is based on obtaining a rough initial estimate of AO and AC peaks by formulating a template from the ensemble average of few initial beats. This rough estimate is then employed to obtain finer estimate by detecting the peaks in the sliding template. For each incoming beat segment, a new sliding template is formulated by the ensemble averaging of the previous few beat segments. The undesired distortions and noise effects are minimized in this process such that the peaks are easier to detect. Further, sliding template aids in avoiding error propagation in case of erroneous peak detection.

## Results

### Experimental Setup and Protocol

The experiments were conducted on 7 subjects aging 28.7 ± 1.89 years with BMI 24.23 ± 3.12. No prior or prevailing heart condition was reported by the subjects. Two postures were considered in this study i.e. lying down flat (supine) and sitting in a chair (seated) as shown in Fig. 2. The supine posture is a usual standard with SCG studies as it limits the unwanted movement of the subjects to some extent as this movement can severely corrupt the SCG signal. Total 19 trials from 7 subjects were recorded in this posture. The seated posture, on the other hand, did provide more real-life scenario. However, it posed added challenge as the subject’s upper body was prone to back and forth movements. Total 9 trials from 3 subjects were recorded in this posture. The duration for each trial was set to 3 minutes and adequate resting period was provided in between the trials.

The experimental setup consisted of recording sternal acceleration (SCG) from accelerometer and ECG simultaneously. One ADXL 327 triple-axis analog accelerometer (Analog Devices) with the range of ±2*g* and sensitivity of 420 *mV*/*g* was placed on the subject’s lower sternum as shown in Fig. 2. This location is commonly reported in the literature. The analog output from the accelerometer was digitized and stored by parallel 16-bit Analog to Digital Converters (ADCs) of Dspace DS1104. For recording ECG, three AgCl electrodes were placed on the subject in Lead-II configuration and the signal was acquired by BIOPAC MP36 (BIOPAC Inc.). The sampling frequencies for both the acquisition devices were set to 500 Hz.

The total number of heart beats/cycles identified for the supine trials and seated trials are 3776 beats and 1868 beats respectively. For evaluation purpose, each cycle was manually annotated.

### Performance Measures

In order to evaluate the performance of the proposed algorithm on the experimental data, following performance metrics were employed:

#### Number of Misclassified Peaks

If the identified peak is not detected within some small variation/threshold of the true peak, then it is considered as misclassified peak. Let **p**_*true*_(*n*) be the true location of the *n*^*th*^ cycle, then **p**_*meas*_(*n*) will be misclassified if |**p**_*true*_(*n*) − **p**_*meas*_(*n*)| > *ζ*, where *ζ* is the threshold that governs the tolerance on variation. *ζ* is set to 2 *ms* or 1 sample in the subsequent analysis. Therefore, the number of such misclassified peaks (*NMP*) is employed as theperformance measure and lower *NMP* is desired.

#### Bland Altman Analysis

Bland Altman (BA) analysis^37^ is performed to check for any bias and systematic errors in the identified peak locations. Further, robustness of the method under test is also evaluated by the Limits of Agreement (LoA) provided by the BA plots. Let **x**_*true*_ be the true peak locations and **x**_*meas*_ be the measured peak locations in the time series, then the measurement error is defined as **x**_*e*_ = **x**_*true*_ − **x**_*meas*_. Hence, LoA can be identified as *mean*(**x**_*e*_) ± 1.96 × *SD*(**x**_*e*_), where *mean*() and *SD*() represents mean and standard deviation respectively.

### AO Detection Performance

BA analysis is performed to evaluate the identification performance of AO peak location for supine and seated trials as shown in Fig. 3(a) and (b) respectively. It is observed that most of the erroroneous detection lie within ±2 ms which correspond to ±1 sample. Further, there exist very few outliers at ±4 ms in the supine condition which correspond to only 0.37% of the cycles. The lower detection error for AO peaks is expected due to less variation in its location with heart rate and its higher magnitude. Since the LoA widths for both the supine and seated cases are sufficiently small, subsequent processing is not required as opposed to the AC peak detection.

### Parametric Analysis

The proposed approach involves formulation of the initial template and sliding template which are governed by the number of beats employed in the process. The number of beats to formulate the initial template and the sliding template are denoted as *N*_*IT*_ and *N*_*SL*_ respectively. *N*_*IT*_ is responsible for the initialization of the algorithm as the quality and peaks location of the initial template depends on the number of beats used. Generally *N*_*IT*_ should be large to provide a good trend of the desired peaks timing, but any discrepancy can be handled by varying the *N*_*SL*_. Therefore in this study, *N*_*IT*_ was set as 60 (which accounts for less than one-minute data in general) and the effects of changing *N*_*SL*_ on the number of missed AC peaks (*NMP*) were analyzed. The other parameters that can potentially influence the correct detection of AC were the tolerances *Tol*_*AC*−*SL*_ and *Tol*_*AC*_ (more details in Methods section) with respect to a reference location in the sliding template and the SCG signal respectively.

To analyze the effects of variation in these parameters, grid search was employed where the objective was to identify the least *NMP*. For this purpose, *NMP* per trial averaged over all subjects was identified for all the three parameters *N*_*SL*_, *Tol*_*AC*−*SL*_ and *Tol*_*AC*_. However, since the resulting search data was 4-dimensional, multiple 3-dimensional surface plots of *NMP* versus *Tol*_*AC*−*SL*_ and *Tol*_*AC*_ were generated for each *N*_*SL*_. However, due to space limitations, only three such plots for *N*_*SL*_ = [1, 15, 40] are illustrated in Fig. 4(a) for supine trials and Fig. 4(b) for seated trials. The aim was to identify the parameters that provide least *NMP*. However, it was observed that the parameter set that provides *NMP* within a small range of global minima was not unique. To identify such multiple parameter sets, the surface was dark shaded if *NMP* is below a particular threshold (small value above global minima) and lightly shaded otherwise. The least *NMP* observed among all *N*_*SL*_ as 3.7895/trial for supine trials with dark shade corresponding to *NMP* < 4.7895/trial. For seated trials, the least *NMP* observed among all *N*_*SL*_ was 11 and the dark shade corresponded to *NMP* < 11.5/trial. It can be observed that for *N*_*SL*_ = 1, the least *NMP* is much greater than the global minima which indicates the absence of the dark region in both supine and seated trials. However, as *N*_*SL*_ was increased, the dark region appeared (e.g. at *N*_*SL*_ = 15) and became larger progressively (e.g. at *N*_*SL*_ = 40) for both supine and seated trials. This implies that the sensitivity of the proposed approach towards change in parameters decreased as *N*_*SL*_ was increased. For each *N*_*SL*_ in the grid search, least *NMP* (local minima) was identified and is illustrated in Fig. 4(b) and (d) for supine and seated trials respectively. The highlighted instances represent the *N*_*SL*_ values for which the 3-dimensional surface plots are illustrated in Fig. 4(a) and (c). It is identified that increase in *N*_*SL*_ tends to decrease the *NMP*, implying better performance.

### Generalized vs Subject-specific Parameters

For each subject, the grid search with parameters *N*_*SL*_, *Tol*_*AC*−*sl*_ and *Tol*_*AC*_ was conducted to identify least *NMP* averaged over all trials for that particular subject. The resulting parameters were termed as subject-specific parameters. Similarly, the generalized parameters were identified as the parameters corresponding to least overall *NMP* (for all subjects). The identified subject-specific parameters are illustrated in Fig. 5(a–f) for supine and seated trials respectively. Similarly, the identified generalized parameters are provided in Fig. 5(g) and (h) for supine and seated trials respectively. From the preceding analysis, it was observed that higher *N*_*SL*_ results in better detection performance. However, higher *N*_*SL*_ corresponds to larger number of beats to be included in the template for annotation of current beat. This implies that the proposed approach should acquire more data before it could start annotating the signal. Hence, the search for *N*_*SL*_ was limited to 40 beats which are approximately 30 seconds of the signal. Further, it can be observed from Fig. 4(b) and (d) that even though the least *NMP* is greater for *N*_*SL*_ = 15 as compared to *N*_*SL*_ = 40, the difference is considerably small. Therefore, both *N*_*SL*_ = 15 and *N*_*SL*_ = 40 were selected for comparison against subject-specific parameters and the corresponding parameter set is called Generalized-15 and Generalized-40 respectively.

Figure 6(a) and (b) illustrate the trial-wise comparison between generalized parameters (with *N*_*SL*_ = 15 and *N*_*SL*_ = 40) and subject-specific optimal parameters for supine trials and seated trials respectively. The absence of bars in trials 4, 5, 6 and 17 for supine condition and trials 1 and 3 in seated condition indicates no missed peak with the generalized and subject-specific parameters. It can be observed that the number of missed peaks is lowest for subject-specific parameters in all the supine trials. Similar was observed for seated trials except for trial 6. The number of missed peaks with generalized-15, generalized-40 and subject-specific parameters were found to be identical for eight trials (trials 2, 4, 5, 6, 9, 10, 12 and 17) in supine condition and two trials (trials 1 and 3) for seated condition. Further, it can be observed that the number of missed peaks with generalized-40 and subject-specific parameters is identical for 15 trials in supine condition and 3 trials in seated condition. The number of missed peaks with generalized-15 parameters is highest 8 supine trials and 4 seated trials. However, except for trial 3 in supine condition, the differences are considerably small.

To statistically quantify the comparison, BA analysis is performed for all the AC peak detection (all subjects and all trials) with generalized-15, generalized-40 and subject-specific parameters for supine and seated trials as illustrated in Fig. 6(c) and (d) respectively. The vertical axis represents the difference between true AC peak locations and the detected AC peak locations, whereas the horizontal axis represents the average of true locations and detected locations. It can be observed that the differences in detected peak locations and the true locations are mostly distributed horizontally with no trend or dependence on the range of values and thus ruling out any systematic error for all three methods for both the supine and seated trials. Further, the mean of the differences in true and detected peak locations with generalized and subject-specific parameters are close to zero which shows unbiased detection performance for both supine and seated trials. The robustness of the detection is associated with the vertical distribution of error and is quantified by the Limits of Agreement (LoA) as illustrated by solid horizontal lines. For better visualization, the width of LoA for peak detection with generalized parameters and subject-specific parameters for both supine and seated trails are illustrated in Fig. 6(e). It can be observed that the performance of the proposed approach is nearly identical for the subject-specific and generalized parameters in supine trials. However, the performance with subject-specific parameters is improved to some extent for the seated conditions. This could be attributed to the difficult nature of peak identification in seated trials due to possible motion or other artifacts as compared to supine trials. Nevertheless, considering the trade-off between identifying subject-specific optimal parameters and improvement in performance, employing generalized-15 parameter set is recommended.

## Discussion

Owing to the complex morphology and inter-subject variability in siesmocardiogram (SCG) signal, detection and annotation of the desired peaks is difficult with the conventional peak detection methods as compared to peak detection in electrocardiogram (ECG) signals. The AO peak is comparatively easier to annotate due to its higher magnitude and lesser variations relative to R-peak of ECG. However, the variations in amplitude and timing of the AC peaks in each heart beat cycle makes its annotation more challenging and requires additional processing. It is known that the cycle-to-cycle difference or the difference of the peak location in consecutive cycles is generally smaller. However, relying only on the peak location of previous cycle may result in erroneous detection in the current cycle or even trigger error propagation if there is incorrect peak detection in the previous cycle.

The proposed approach solves these issues by first identifying the desired peaks in the ensemble-average based sliding template and then employing this information to search for the desired peak in actual SCG signal in the vicinity. The template contains less noise and the surrounding undesired peaks are mitigated, making it easier to identify the desired peaks in the template. This approach has three free parameters: number of beats in sliding template, search window width in the template and search window width in the SCG signal. However, it is observed from the results that the proposed approach becomes less sensitive to the parameters if more number of beats are included in the sliding template as the range of other parameters increase that can provide similar detection performance. This is evident from the similar detection performance between generalized and subject-specific parameters. Since the range/choice for selecting the generalized parameters was large, the proposed approach does not require extensive search or training to identify the parameters that provide adequate detection performance.

The overall detection performance of the proposed approach increased by incorporating more number of beats in the sliding template, hence requiring more cycles before annotation can be started. Therefore, there exists a trade-off between maximizing the performance and minimizing the cycles required before annotation. However, the results indicate that the performance difference between sliding template with 15 beats (Generalized-15) and 40 beats (Generalized-40) is insignificant (as observed in Fig. 6(e)).

The segmentation of the SCG and ECG signals in the proposed approach depends on the reliable detection of R-peak of ECG. Although the PanTompkins method^38^ reliably detected the R-peak locations, however, several signal processing techniques have been developed in last few decades and one can employ any other technique based on the feasibility. For further details on the implementation and analysis of the segmentation, readers can refer to the supplementary material. In future, we are also considering beat detection in SCG signals in which the AO peak detection can be made partially independent of the ECG signal. Hence in conditions where R-peak detection is difficult, independent AO peak detection can be utilized and vice versa. The fixed segment length selection before the annotation process can handle normal variations in the heart rate and does not force any desired peak outside the segment. However, different activities and emotional reactions can lead to sudden deviation of heart rate which is a limitation for this approach and therefore adaptation of segment length may be required by constant tracking of the R-R intervals which will be addressed in future work.

Recently, an attempt was made to automate the annotation process with envelope based method^23^. However, the peaks in the SCG signal are searched backward from the corresponding envelope peak which may limit the performance in the presence of additional peak(s) due to artifacts. The LoA widths for envelope based approach in supine and seated trials are identified as 38.2 ms and 62.5 ms respectively. Whereas, the LoA widths of the proposed approach with generalized-15 parameters in supine and seated trials are 16.1 ms and 42.9 ms respectively, which are significantly smaller than the existing approach. This implies better AC peak detection performance of the proposed approach.

The robustness of the proposed approach was tested by analysis of peak detection performance under noise. Gaussian noise was introduced in the SCG signals in two different scenarios: i) pre-filtering noise (noise added before filtering) and ii) post-filtering noise (noise added after filtering). Figure 7 shows the effect on the peak detection accuracy of the proposed approach (with generalized-15 and generalized-40 parameters) and the envelope based method for all the trials under various levels of Gaussian noise with the standard box plot. The peak detection accuracy represents the number of peaks detected within ±2 ms or ±1 sample of the actual peak. It is observed that the AC peak detection accuracy is similar in all cases for generalized-15 and generalized-40 parameters of the proposed approach, which is also confirmed by Wilcoxon signed rank test. The proposed approach outperforms the envelope based method, especially in the post-filtering noise scenarios as shown in Fig. 7(b) and (d). A significant difference between the medians of detection accuracy with proposed and envelope based methods was observed for supine trials in both pre-filtering and post-filtering noise scenarios (*p* < 0.01). For seated trials, shown in Fig. 7(c) and (d), the medians of the detection accuracies for the proposed and the envelope based approach are similar for higher SNRs, but the inter-quartile range of the detection accuracy with proposed approach is lower than the envelope based approach for almost all the values of SNR, implying its robustness. Similar analysis is performed for AO peak detection accuracy with the proposed approach as shown in Fig. 7(e) and (f). It is observed that even for the worst case (0 dB SNR), the AO detection accuracy is higher than the AC detection accuracy for the given conditions (noise type and trial type).

Owing to the potential advantages of personalized health monitoring systems, efforts are being made to develop wearable prototypes for acquiring SCG and ECG signals. However, the consumer may not benefit from such systems unless a quantified measure of health status is provided. The proposed approach could be employed to alleviate this problem as the STI measurements can provide such quantified measure. Further, most of the research on SCG signals is focused on supine posture as the body movements and other artifacts are minimized. However, for extracting information from SCG in wearable configuration, real-life scenarios/postures should also be evaluated. For this purpose, seated posture is considered in this study for preliminary investigation as most of the time is spent in this posture in daily routine. However, due to lesser restrictions on body movement, annotation of seated trials are more challenging as compared to the supine trials and the same is confirmed by the experimental results. Therefore, more improvement is required to alleviate these challenges. Evaluation of proposed approach on additional postures and routine tasks is not considered in this paper as motion artifacts corrupt the signal heavily, rendering the SCG signal unreliable. Further, high amplitude momentary artifacts such as hiccups that occur for one or few beats can corrupt the sliding template and can result in unreliable annotation of few consecutive SCG beats. Therefore, the future work will be focused on developing methods to remove these artifacts as well as to improve the template formulation. One potential solution is to employ blind source separation based technique for artifact removal^39^. However, motion artifact removal is quite challenging and will be addressed in future. Artifact removal prior to annotation may result in increased detection performance for the supine and seated conditions and inclusion of new postures may also be possible.

## Methods

The proposed approach was validated on the SCG and ECG signals recorded from 7 human subjects with their informed consent. These experiments were approved by the institutional review board of Kyungpook National University and were performed in accordance with the principles expressed in the declaration of Helsinki.

### Preprocessing

The high-frequency noise and baseline wander in the raw SCG and ECG signals were removed by bandpass filtering. A 5^*th*^ order Butterworth filter with the passband of [1 35] Hz^23^,^40^ and [1 100] Hz^41^ was employed for SCG and ECG respectively. The same filter orders were used for both SCG and ECG signals to avoid discrepancy in the latencies between the two signals due to filtering.

Figure 8 illustrates the flow diagram of the proposed approach. Two stages of template formulation are employed in the annotation process: Initial template and Sliding template. The initial template aids in the direct detection of AO peaks (systolic peak) in the SCG signal as well as diastolic peaks in the sliding template, which further aids in the detection of diastolic peaks in the SCG signal.

### Initial Template Formulation

**Figure 1:**
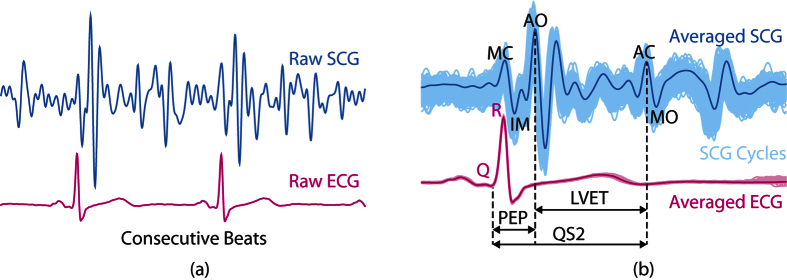
SCG vs. ECG signal. (**a**) Consecutive SCG and ECG Cycles (**b**) superimposed SCG and ECG beats (light shades) aligned w.r.t R-peaks and their ensemble averages (dark lines).

**Figure 2:**
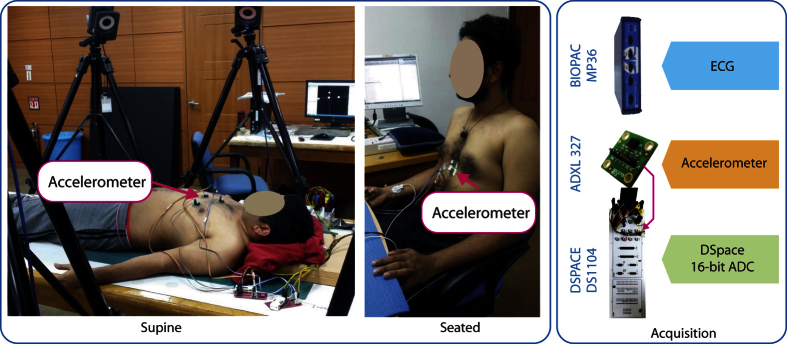
Experimental Setup - postures and data acquisition. Accelerometer placed at lower sternum was considered for this study.

**Figure 3:**
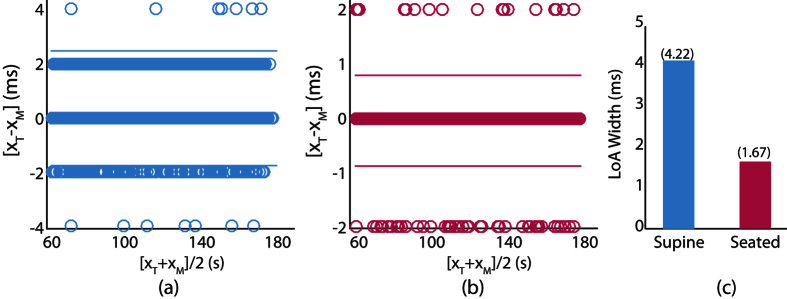
BA plots for AO peaks for supine trials (**a**) and seated trials (**b**) with LoA widths for both conditions (**c**). *X*_*T*_ is the true location while *X*_*M*_ is the identified location of AO peak by proposed approach.

**Figure 4:**
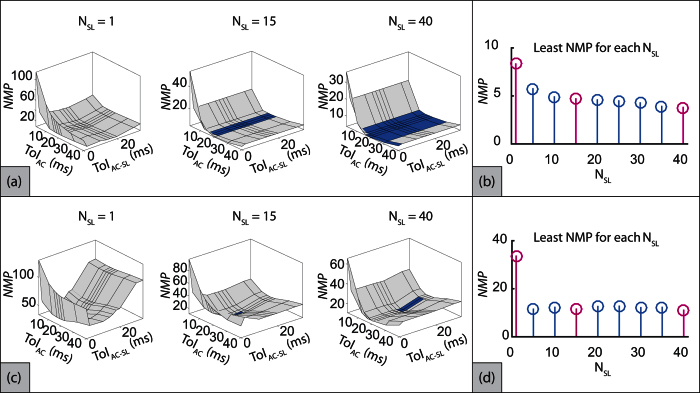
Optimal *N*_*SL*_, *Tol*_*AC*−*sl*_ and *Tol*_*AC*_ w.r.t. number of misclassified peaks for supine trials. Dark shade shows region where 3.7895 ≤ *NMP* ≥ 4.7895 and light region shows *NMP* > 4.7895.

**Figure 5:**
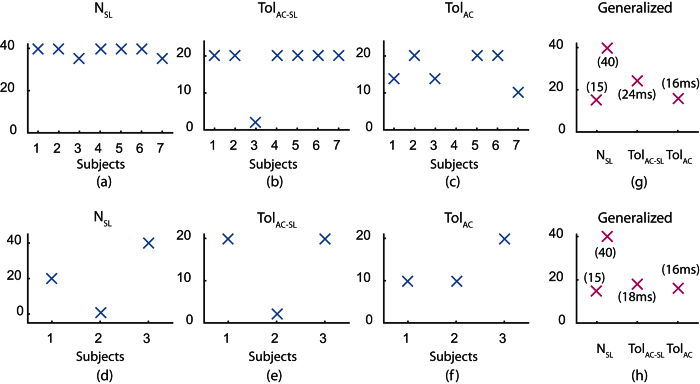
Subject-specific optimal parameters (**a**–**f**) and generalized optimal parameters (**g**, **h**). Subplots (**a**–**c**) and (**f**) correspond to supine trials while (**d**–**f**) and (**h**) correspond to seated trials.

**Figure 6:**
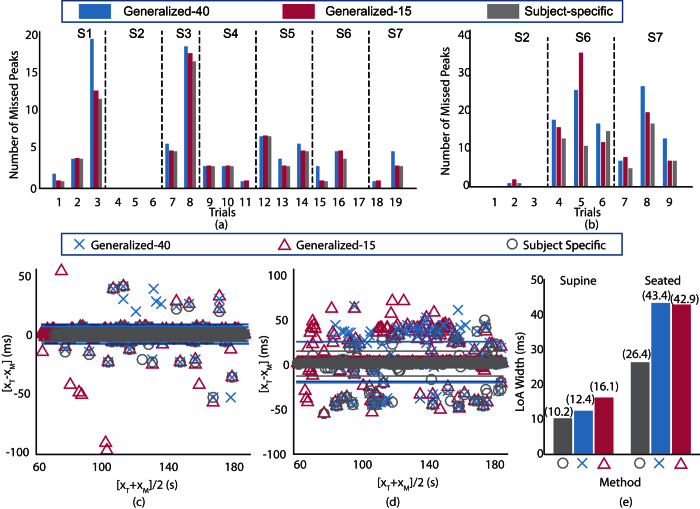
Comparison between detection performance of generalized parameters and subject specific parameters. Number of misclassified peaks with generalized parameters and subject-specific parameters for supine trials (**a**) and seated trials (**b**). BA analysis of annotation error for supine trials (**c**) and seated trials (**d**) with the limits of agreements for both conditions (**e**).

**Figure 7:**
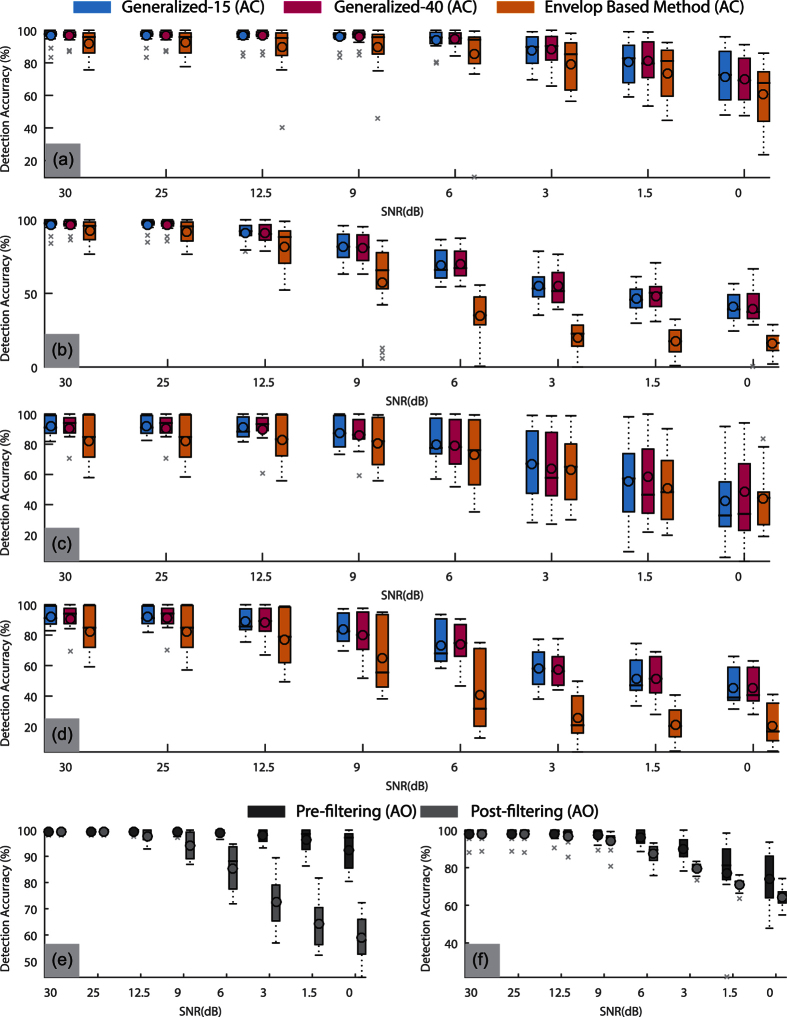
Demonstration of the effect of varying levels of Gaussian noise on AO/AC peak detection accuracy with box plots. (**a**) AC detection accuracy in supine trials with pre-filtering noise, (**b**) AC detection accuracy in supine trials with post-filtering noise, (**c**) AC detection accuracy in seated trials with pre-filtering noise (**d**) AC detection accuracy in seated trials with post-filtering noise, (**e**) AO detection accuracy in supine trials with both pre-filtering and post-filtering noise and (**f**) AO detection accuracy in seated trials with pre-filtering and post-filtering noise.

**Figure 8:**
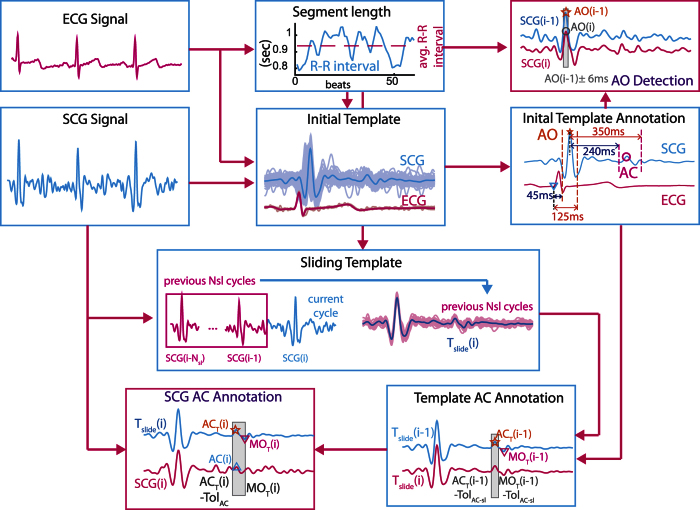
Proposed Approach.

Initial template formulation is an offline procedure which provides the estimate of the location of the desired systolic and diastolic peaks in the upcoming SCG signal for initialization. To form the initial template, the first few beats (*N*_*IT*_) of the SCG signal are considered. The R-peaks of the corresponding ECG signal are detected using Pan-Tompkins method^38^ as shown in Fig. 1(a). Then both the SCG and ECG signals are segmented and aligned such that the R-peak for each beat occurs at a specific location (e.g. at 20% of the segment length) in each segment as shown in Fig. 1(b). Let **r** be the vector containing the location of R-peaks, *w* be the length of segment and *s* be the location specifier of the R-peaks in segment, then the current segment (**Seg**_**SCG**−**R**_^(*i*)^) is defined as:


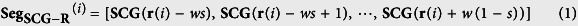


where **Seg**_**SCG**−**R**_^(*i*)^ represents the SCG segment. The length of these segments *w* is fixed as the median of R-R interval for the first (*N*_*IT*_) beats, whereas *s* is set as 0.2. Therefore, each segment initiates at *s* × *w* samples prior to the R-peak location and terminates (1 − *s*) × *w* − 1 samples after the R-peak location of the current beat. Hence the R peak occurs at (*s* × *w* + 1)^*th*^ sample in every segment. The initial template for the SCG signal is then formulated by ensemble averaging all the segments of SCG signal such that


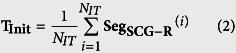


Ensemble averaging aids in suppressing the uncorrelated noise and distortions, while the consistent peaks (i.e. peaks of interest) are enhanced.

The annotation of AO and AC peaks in the initial template signal is fairly easy and can be performed either manually or with simple automation. In this study, we employed automated procedure for this purpose. The AO peak in the initial template i.e. AO_*IT*_ is identified as the highest peak in a relaxed Pre-Ejection Period (PEP) range i.e. [45 120] *ms* interval from the Q-peak of ECG template. Similarly, the diastolic peaks i.e. AC_*IT*_ and MO_*IT*_ are identified as the highest peak and lowest valley in the interval of [240 350] *ms* from AO_*IT*_. This interval is selected as a relaxed range for Left Ventricular Ejection Time (LVET). The locations for AO_*IT*_, AC_*IT*_ and MO_*IT*_ are stored for the subsequent processing.

### AO Peak Detection

Due to less variation of PEP as compared to LVET in consecutive beats^24^, detection of AO peak in SCG signal is fairly simple and does not require any further processing of the signal. The first AO peak of the SCG under annotation (other than that used for initial template formulation) is identified as the maxima in the vicinity of the AO peak location in initial template. Therefore, the search window is defined as [AO_*IT*_ ± AO_*Tol*_]. The next peak **AO**(*i*) is identified as the maxima in the vicinity of the previous peak **AO**(*i* − 1) and hence the search window is defined as [**AO**(*i* − 1) ± AO_*Tol*_]. Similarly, all the subsequent AO peaks are annotated.

### Diastolic Peaks Detection

Since diastolic peaks are smaller in magnitude, these are prone to noise and artifacts. Therefore, we use sliding template to minimize the effects of noise and artifacts.

#### Sliding Template

The location of the desired peaks can change over time and become too distant from the estimate provided by the initial template. If this estimate is updated without the sliding template, then error propagation can occur as the peak detection for current beat will depend on previous peak location. To avoid error propagation, while minimizing the effects of noise and distortion, sliding template methodology is developed. Due to varying distance between the corresponding R-peak and AO-peak in each beat, the SCG segments are aligned such that AO peak occurred at a specified location in each segment. The segmentation is performed as


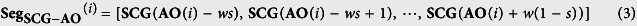


where **AO** is a vector containing the locations of AO peak in the SCG signal. To annotate the current (*i*^*th*^) segment, the ensemble is formed with the previous *N*_*sl*_ number of segments. Therefore, the sliding template for the *i*^*th*^ beat is defined as:


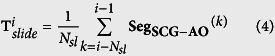


Before identifying the locations of AC and MO peaks in the SCG signal, the AC and MO peaks in the corresponding sliding template that are denoted as **AC**_**T**_ and **MO**_**T**_ respectively. **AC**_**T**_(*i*) is identified as maxima in the search window [**AC**_**T**_(*i* − 1) − *Tol*_*AC*−*sl*_**MO**_**T**_(*i* − 1) − *Tol*_*MO*−*sl*_]. Similarly, the **MO**_**T**_(*i*) is identified as the minima in the search window [**AC**_**T**_(*i*)**MO**_**T**_(*i* − 1) + *Tol*_*MO*−*sl*_].

Intuitively, the search windows for **AC**_**T**_(*i*) and **MO**_**T**_(*i*) can be simply **AC**_**T**_(*i* − 1) ± *Tol*_*AC*−*sl*_ and **MO**_**T**_(*i* − 1) ± *Tol*_*MO*−*sl*_. However, since the mitral valve opens after the atrial valve closure, **MO**_**T**_(*i*) never precedes **AC**_**T**_(*i*). Therefore, the bounded search windows serve dual-purpose: i) shorter search window implies increased computational efficiency and ii) shorter window is less prone to the erroneous detection of the desired peaks.

The initialization of the search windows for diastolic peaks in the template i.e. **AC**_**T**_**(0)** and **MO**_**T**_**(0)** is performed using the peak locations from the initial template. Let *LVET*_*IT*_ be the LVET of initial template i.e. *LVET*_*IT*_ = *AC*_*IT*_ − *AO*_*IT*_ then **AC**_**T**_**(0)** is defined as **AC**_**T**_**(0)** = *AO*_*T*_ + *LVET*_*IT*_, where *AO*_*T*_ is the location of AO peak in template. Note that *AO*_*T*_ remains constant for all the segments due to the segmentation procedure as shown in Eqn 3. Similarly, **MO**_**T**_**(0)** is defined as **MO**_**T**_**(0)** = **AC**_**T**_**(0)** + (*MO*_*IT*_ − *AC*_*IT*_).

#### Peaks Refinement

The AC and MO peaks in the SCG signal became fairly easier due to even shorted search window once the location of the AC and MO peak in the corresponding sliding template is identified. Let **AC**(*i*) be the location of AC peak in the SCG signal, then it is identified as the maxima in the search window [**AC**_**T**_(*i*) − *Tol*_*AC*_, **MO**_**T**_(*i*)]. Similarly, the MO peak in the SCG signal is identified as the minima in the search window [**AO**_**T**_(*i*), **MO**_**T**_(*i*) + *Tol*_*MO*_].

Since the signal in the sliding template is much cleaner than the actual SCG signal, therefore is less prone to detecting the erroneous peaks. This helps in avoiding the error propagation. For instance, if the SCG signal itself is used instead of the sliding template, the current peak in the SCG signal will depend on the correct detection of the previous peak. If there is an error, it will shift the search window for the next peak which may result to the erroneous detection of next peak. However, in the proposed approach, even if peak in one beat is detected erroneously, the next peak will depend on the average of previous few peaks in the formation of sliding template.

## Additional Information

**How to cite this article**: Shafiq, G. *et al*. Automatic Identification of Systolic Time Intervals in Seismocardiogram. *Sci. Rep*. **6**, 37524; doi: 10.1038/srep37524 (2016).

**Publisher’s note:** Springer Nature remains neutral with regard to jurisdictional claims in published maps and institutional affiliations.

## Supplementary Material

Supplementary Information

Supplementary Video S1
