# Supplementary material for: Automatic Identification of Systolic Time Intervals in Seismocardiogram
Source: Sci Rep. 2016 Nov 22;6:37524. doi: 10.1038/srep37524 (PMC5118745; doi:10.1038/srep37524)
Supplement: Supplementary Information [file srep37524-s1.pdf]

Supplementary Material: Automatic Identification of Systolic Time Intervals in Seismocardiogram

G. Shafiq[1], **S. Tatinati**[1], **Wei Tech Ang**[2] **and K. C. Veluvolu**[1,2*]

[1] School of Electronics Engineering, Kyungpook National University, Daegu, South Korea 702-701
[2] School of Mechanical and Aerospace Engineering, Nanyang Technological University, Singapore 639798
(e-mail*: veluvolu@ee.knu.ac.kr)

ABSTRACT

In this supplementary information material, additional details on the iterative implementation of the segmentation procedure for ECG signal is explained. First it is shown how the segmentation depends on the R-peak detection and then we demonstrate that inclusion of all PQRST peaks is not required for the annotation of the SCG signal. The segmentation strategy is analyzed for high variations in R-R intervals in the ECG signal, and the reliability of the segmentation strategy under such conditions is demonstrated. Further, as an additional robust measure, an automated elimination procedure for the beats/segments with high variations is discussed which minimizes the chances of corrupting the sliding template.

Automated Segmentation Strategy

The segment having all PQRST peaks is not required for annotation of the SCG signal. To achieve the objective of this work i.e. annotation of AO and AC peaks of the SCG signal, only identification of R-peak is required. The ECG and SCG segments can be simply formulated with certain samples (20% of segment length) before and certain samples (80% of segment length) after the occurrence of the R-peak. The R-R interval can vary significantly during the arrhythmias (e.g. PVC) and with the fixed segment length, the consecutive segments can be disjoint or partially overlap depending on the changing R-R interval as shown in Fig. S1. Analysis on such trials from MIT-BIH arrhythmia database is performed to demonstrate how the fixed segmentation strategy copes up with the variations in the ECG signal. Further, it is demonstrated that the beats with sudden variations can be either used or eliminated from both the annotation and sliding template formulation processes in automated manner.

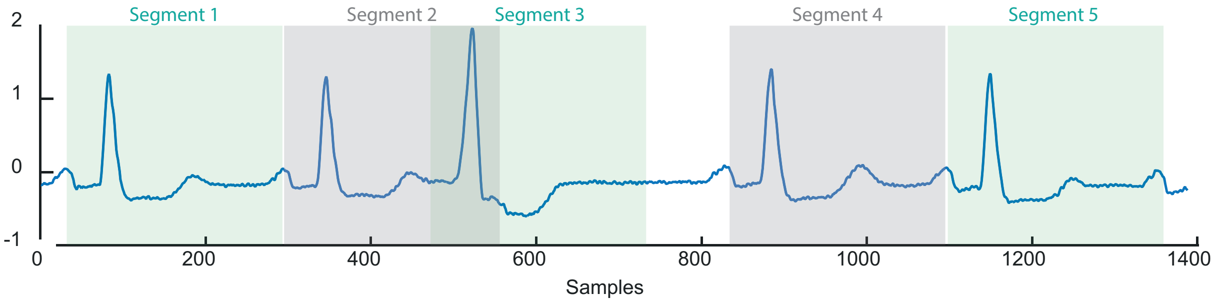

Figure S1. ECG time series and consecutive segments

The automated segmentation strategy employed in this work depends on reliable detection of ECG R-peak in an iterative manner. Therefore, the R-peak detection procedure is briefly explained that will aid in understanding the automated segmentation process which will be explained subsequently.

Real-Time R-peak Detection

For real-time ECG-R peak detection, we have employed the existing algorithm developed by Pan-Tompkins.[N1] Fig. S2 shows the flow-chart for ECG R-peak detection. The raw ECG signal is filtered, differentiated with 5-point derivative, transformed by squaring and moving average filter (150ms) is applied. All these processes are possible in an iterative manner with integer arithmetics and are feasible in real-time (with some processing delay).[N1] For example, filtering and differentiation are done by real-time convolution of the filter response with the incoming signal or with the equivalent difference equations.

The peaks in the integrated signal ECG_{MA} are detected by sensing the slope change in the signal as shown in Fig. S2. Note that this peak is not the actual R-peak in ECG and subsequent processing will be performed. Physiologically, the R-R

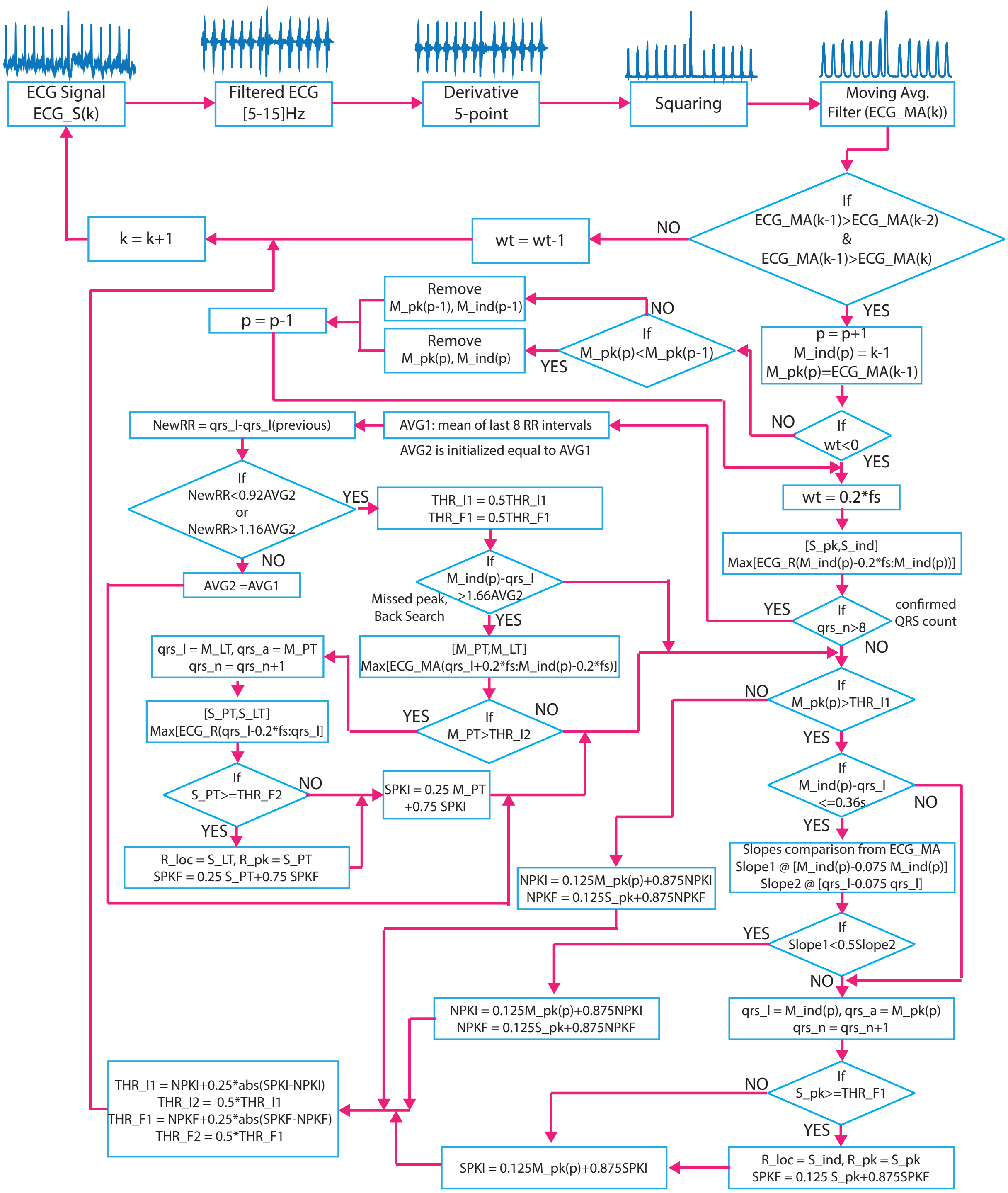

Figure S2. Flow-Chart of Automated R-peak Detection Algorithm in Real-Time (Pan-Tompkins).

interval cannot be less than 200ms, therefore the algorithm waits for 200ms and checks if any other peak is detected in this duration. If the detected peaks are within this refractory period, peak with smaller magnitude is discarded. This stage is the major contributor to the delay since algorithm has to wait for 200ms after detecting peak to confirm it. Once, the peak in the integrated signal is decided, a maxima is searched in the raw ECG signal within 200ms of the detected peak. The detected peak in ECG_{MA} is compared with the adaptive threshold. These thresholds are adapted based on peak detection and their amplitude as shown in Fig. S2. The peak in ECG_{MA} exceeding the threshold is checked for potential candidate for T-wave if it occurs within 360ms of the previously confirmed peak in the integrated signal. If the slope at current peak is less than half of the slope

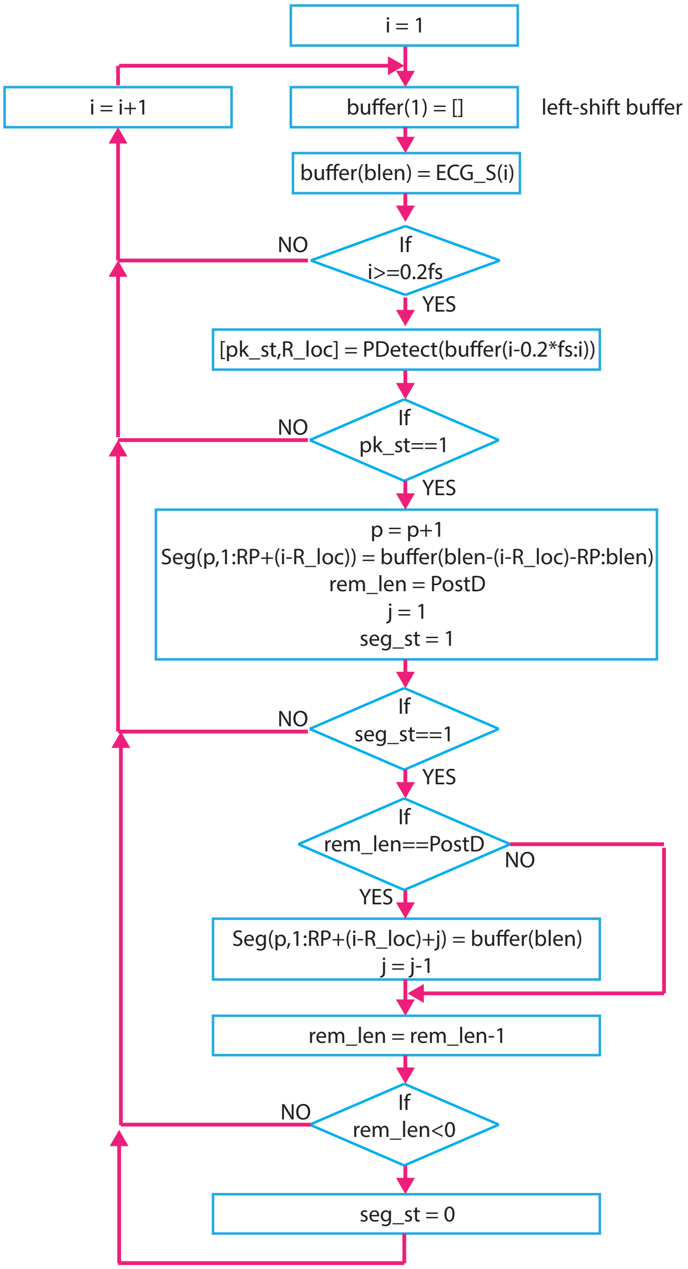

Figure S3. On-line Automated Segmentation Scheme for the Proposed Approach

at previously confirmed peak, then the current peak is identified as T-wave and the thresholds are updated. Otherwise, the peak is confirmed as the qrs complex indicator, and the local maxima in raw signal is compared with the corresponding threshold. The peak in raw signal exceeding the threshold is confirmed as R-peak and the corresponding thresholds are updated.

It should be noted that the algorithm can confirm the R-peak after 300-400ms of its occurrence (200ms for peak confirmation in integration signal and 100-200ms for processing delay at output of moving average filter). A buffer is required for storing few previous samples of ECG for comparison. For example difference between the current sample k and recently detected peak in integration signal $M_{ind}(p)$ can be upto 200ms. Further, the corresponding maxima in the raw signal is also searched within the same 200ms window. Hence, a buffer of only 200ms length is used only for the R-peak detection, which is quite small. Once confirmation and location of the R-peak is received from this detector, the segmentation process can be started.

Automated Iterative Segmentation

The requirements for successful segmentation is that in each segment, at least one R-peak should occur at a pre-specified location (20% of segment length), and the segment length should be sufficient enough to accommodate inclusion of the desired (systolic) peaks in the corresponding Siesmocardiogram (SCG) signal in each segment. The shorter systolic phase (less than 50% of heart beat cycle)[N3] provides sufficient margin for variations in R-R intervals with fixed segment length. Since the peaks of interest are AO and AC peaks which occur after the R-peak in ECG, it is not required to detect and include the P-wave, or make the consecutive segments continuous. As mentioned above, due to fixed segment length, the segments can be disjoint or can partially overlap (as shown in Fig. S1). Furthermore, it should be noted that first the R-peak is detected in the ECG signal and then segmentation is performed. The segmentation process will not begin if the R-peak is not detected.

The iterative R-peak detection algorithm in Fig. S2 can be represented as a function "PDetect" that returns a status flag if peak is detected and the corresponding peak location for each iteration as shown in Fig. S3. Further, a buffer is required to store few samples (blen) of the signal. This buffer serves two purposes: i) provides 200ms buffer for the R-peak detection algorithm and ii) provides enough storage for the signal such that R-peak and few prior samples are available even after the detection processing delay as described earlier. The minimum length requirement for the buffer is $blen_{min} = (i - R\_loc) + RP$, where $(i - R\_loc)$ is the detection processing delay (difference between current sample and location of the latest confirmed R-peak) and RP is the desired location of R-peak in the segment which is to be formulated. Since the desired AO and AC peaks in the SCG signal occur after R peaks, we can start the segmentation from the R-wave i.e. $RP = 1$, minimizing the buffer size requirement. However, for consistency and future consideration of MO peak in SCG (which occur just before R-peak) as well as the Q-peak of ECG for PEP calculations, we set $RP = 0.2 \times RR_{init}$, i.e. 20% of the median R-R interval obtained in formulating the initial template (which is an offline procedure). Therefore, the buffer size required for our application is around 600ms, which was used in the segmentation process.

The automated iterative segmentation process is shown in Fig. S3. The procedure buffers for initial 200ms of ECG data such that peak detection can be started. With each of the incoming ECG sample, the buffer is updated and the latest 200ms of ECG signal is given to the R-peak detection algorithm (as shown in Fig. S2. At each iteration, the algorithm returns $pk_{st} = 0$ if no R-peak is confirmed, otherwise sets $pk_{st} = 1$ and gives the location of currently confirmed R-peak as R_loc. If the peak is detected, then latest $(i - R\_loc) + RP$ samples are taken from the buffer for formulating the initial $RP + (i - R\_loc)$ samples of the segment. The remaining number of samples to complete the segment rem_len is updated. In the subsequent rem_len iterations, the incoming sample of the ECG signal (latest updated sample in the buffer) is included in the segment. An additional animated gif file is provided as the supplementary file to this paper that shows the update process of buffer, R-peak detection, current segment and the previous segment in an iterative manner. One frame from such animation is shown in Fig. S4. The black trace shown with the buffer trace represents peak detection flag pk_{st} at the corresponding instant. After the R-peak occurs and is confirmed, the black trace shows peak at the detected R-peak location.

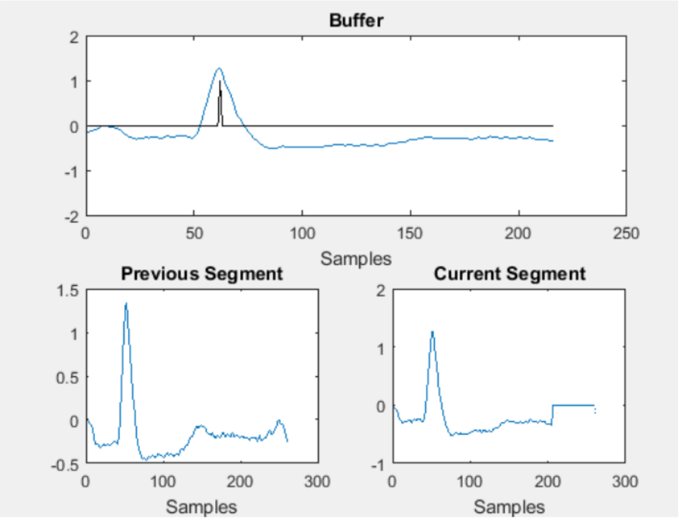

Figure S4. Video Legend (Segmentation Demonstration.gif): Animated gif file that shows the update process of buffer, R-peak detection, current segment and the previous segment in an iterative manner. The black trace shown with the buffer trace represents peak detection flag pk_{st} at the corresponding instant. After the R-peak occurs and is confirmed, the black trace shows peak at the detected R-peak location.

Effects of the Variations in ECG segment

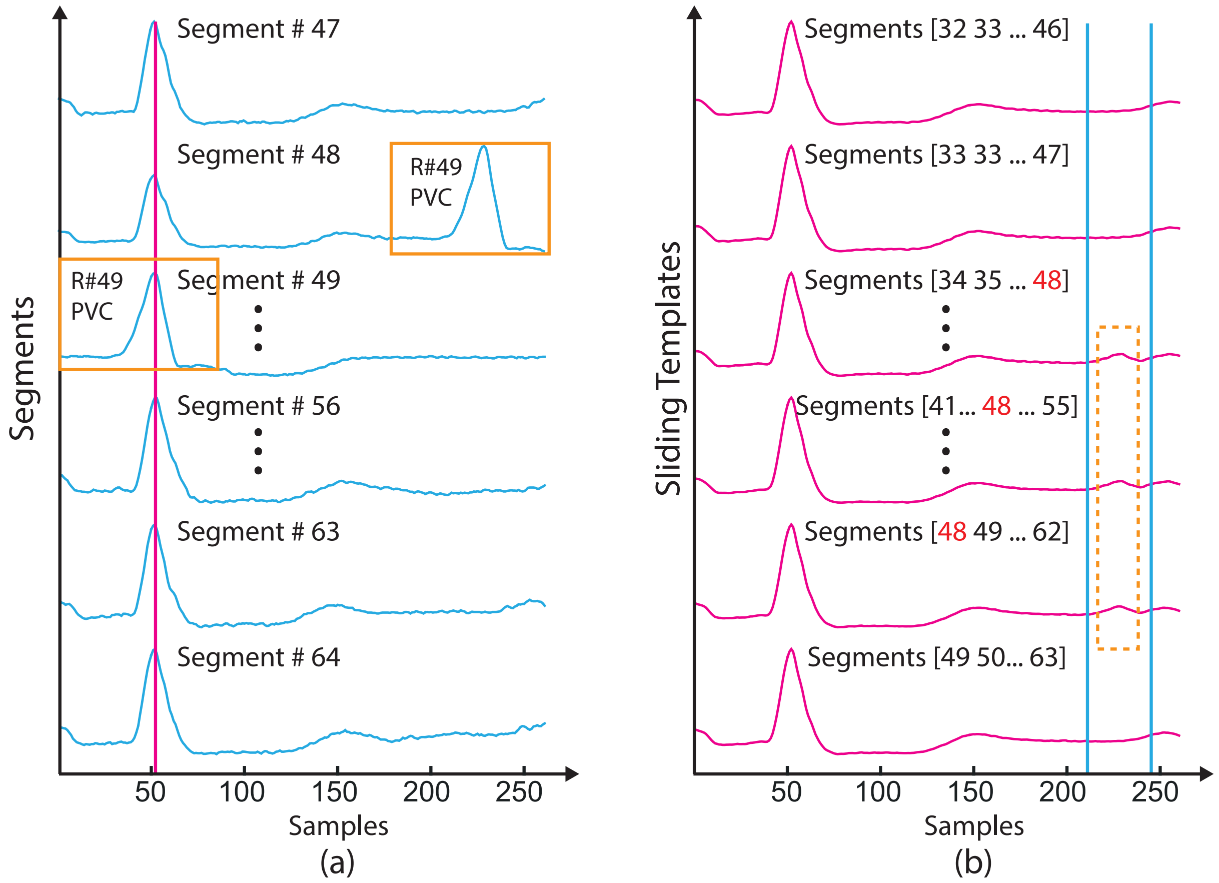

Figure S5. Result of Segmentation with the proposed approach before PVC beat, with PVC beat and after PVC beat

The variations in the ECG segments can be caused by sudden changes in the R-R interval and changes in the morphology (due to artifact or underlying physiological condition). To test the segmentation strategy on the ECG signals with high variations, few trials from MIT-BIH arrhythmia database[N2] were taken. High variations were observed in the Premature Ventricular Contraction (PVC) beats in which the R-peak occurs quite sooner than the normal, resulting in sudden and significant drop in the R-R interval and then significant increase in the R-R interval for the next beat. The aim is to demonstrate that the segmentation strategy and the subsequent steps (formulation of sliding template) can be performed under such extreme variations in the R-R intervals. For illustration, a typical PVC trial from the same database having few episodes of PVC was segmented with the iterative segmentation strategy and is shown in Fig. S3. Few of the resulting segments and their corresponding sliding templates are shown in Fig. S5(a) and Fig. S5(b) respectively. Each segment generally consists of one R-peak per segment, occurring at 51th sample ($RP = 0.2 \times RR_{avg}$) as indicated by vertical line. However, due to PVC, the 49th R-peak (R#49) is also included in the segment #48. Since the R-peak detection is performed prior to the segmentation (for each segment), it is known that the R-peak corresponding to segment #48 is R#48 and from this reference onwards, the subsequent processing will be performed. Note that during the segmentation process, peak detection and buffer update are not stopped and as soon as R#49 is confirmed, new segmentation is started from the buffer. The region indicated by box is the overlapping region between the two segments. Note that this segment is still usable since our region of interest is from R-peak to the T-wave (till around 200th sample). However this beat can also be skipped in an automated manner as demonstrated in the subsequent section of this material.

The sliding templates for the segmented beats are also shown in Fig. S5 (b) to demonstrate the effects of high variations in R-R interval. For each nth segment shown Fig. S5 (a), its corresponding sliding template is formulated from segments $[n - n_{slide}, n - n_{slide} + 1, \cdots, n - 1]$. It is observed that the sliding templates with the segment #48 (PVC) have a small bump in the far-end region as indicated by the vertical lines. However these templates are quite consistent with the normal templates (without segment #48) in our region of interest i.e. AC peaks in SCG segment which generally lie between the peak of T-wave and end of T-wave (between 150-200 samples) are less likely to be disturbed and normal annotation steps can be performed

without requiring additional processing. The effect of PVC on SCG signals and how it affects the identification of AO and AC peaks in SCG will be our future work.

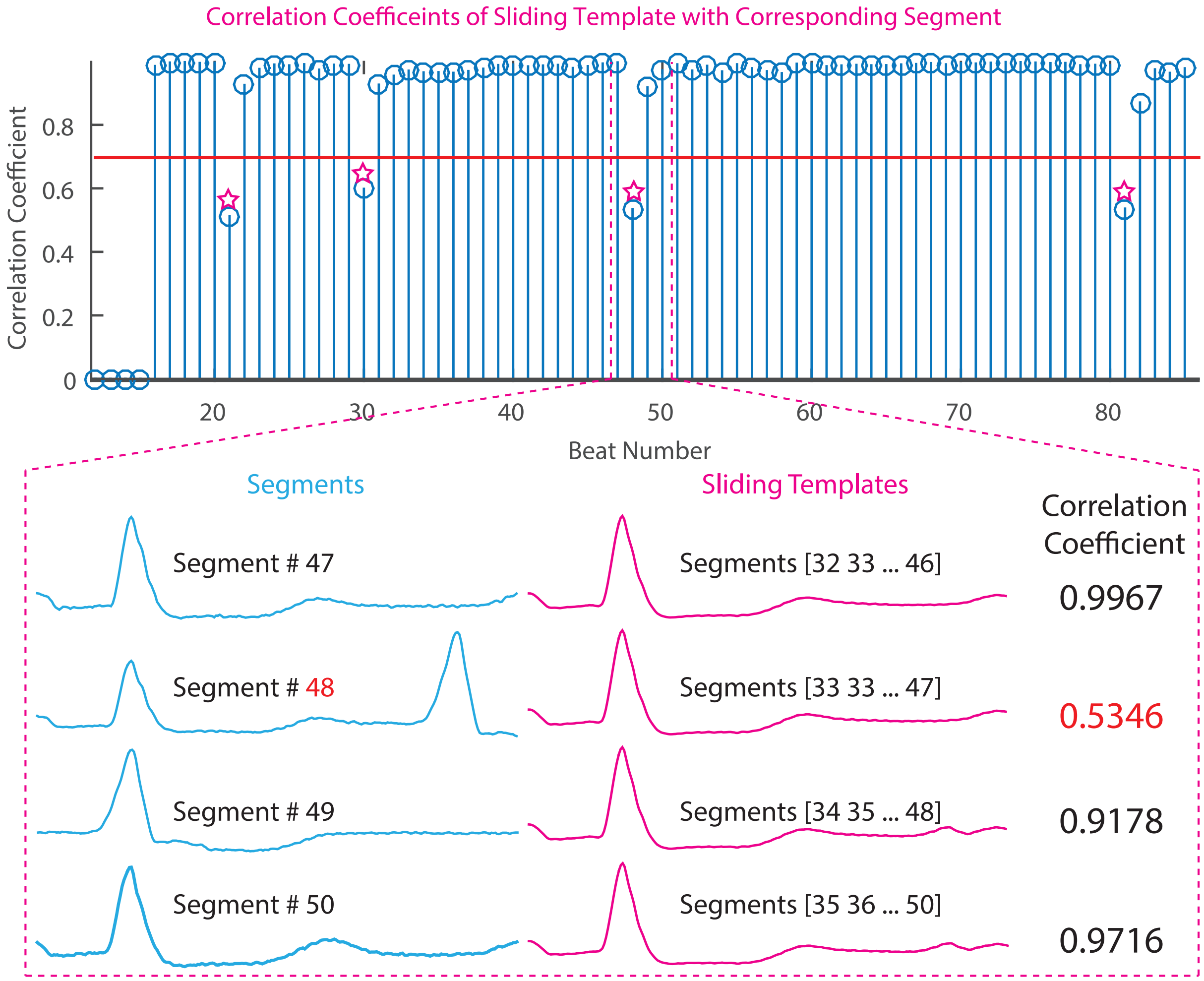

Figure S6. Correlation Coefficients for the Sliding template and the corresponding beat segment

However, to ensure higher degree of reliability, the PVC segment (or any other abnormal segment) can also be excluded from the sliding template. This can be performed by identifying the correlation coefficient of the current beat segment n with its corresponding sliding template formulated from segments $[n-n_{slide}, n-n_{slide}+1, \cdots, n-1]$. Such correlation coefficients are shown in Fig. S6 for first few segments of MIT-BIH arrhythmia trial 105. It can be observed that when a segment with PVC beat comes (as indicated by star marker), the correlation coefficient drops significantly. For illustration, we have chosen one PVC episode (segment#48) and presented the segments 47-50 with their sliding templates and the correlation coefficient identified between them. The correlation coefficient drops from 0.9967 to 0.5346 for the PVC segment and goes back up to 0.9178 and 0.9716 for normal segments. Hence the PVC can be detected in automated manner when the correlation coefficient drops below a certain threshold (e.g. a constant threshold of 0.7 indicated by red line) and therefore the annotation of the segment with PVC can be skipped as well as the PVC segment can be barred from the ensemble for the sliding template. Threshold adjustment and adaptation will be addressed in the future.

Future Considerations for Hardware Implementation

Considering these limitations in hardware, changing segment length with each beat is not necessary in our application. The static segmentation strategy avoids additional computations in deciding and adapting to the segment length continuously. The segmentation of ECG with extreme R-R variations (PVC beat) has been demonstrated and even if an additional peak occurred, no significant effects were observed in our region of interest. Note that the consecutive segments can be disjoint or partially overlap and we do not have any requirement on the continuity of the consecutive segments. Further, in this application, we deal

only with the systolic phase of the heart beat cycle which is smaller than the diastolic phase (less than half of the complete cycle).[N3] Therefore, R-R intervals that are longer than the set segment length (e.g. in case of post-PVC beat) can also be accommodated.

The buffer employed in the segmentation process is fixed to 600ms or 300 samples length at 500Hz, which is quite small. Further, the segmentation process is quite simple and no complex operations are performed. Considering the memory aspect of hardware, each sample will require 4bytes if assigned float variable (6 decimal point precision). The total number of samples to be stored are $s_{total} = s_{buffer} + n_{slide} + 1 \times s_{seg}$, where $s_{buffer} = 300$ is the number of samples required for buffer and $n_{slide} = 500$ is the number of samples required for 1 second segment length at 500Hz, while n_{slide} is the number of beats to be included in sliding template. Then the total number of samples s_{total} becomes 8.3 ksamples requiring $8300 \times 4 = 33200$ bytes or 33.2 kBytes of storage, which is practically feasible for the embedded systems application. However, this procedure is not yet tested and in future we are planning to implement this annotation approach on android platforms for possible integration with smart-watches or mobile phones and any limitations will be addressed there.

References

N1. Pan, J., & Tompkins. W. A Real-Time QRS Detection Algorithm. *IEEE Transactions on Biomedical Engineering*, **32(3)**, pp. 230–236 (1985).

N2. Goldberger, A., L. *et al.* PhysioBank, PhysioToolkit, and PhysioNet: Components of a New Research Resource for Complex Physiologic Signals. *Circulation*, **101(23)**, e215–220 [Circulation Electronic Pages; http://circ.ahajournals.org/cgi/content/full/101/23/e215] (2000).

N3. Hademenos, G. J., and Massoud, T. F. The Physics of Cerebrovascular Diseases: Biophysical Mechanisms of Development, Diagnosis and Therapy. *Springer Science & Business Media*, Chapter 3 (1997).
