# Supplementary figures and images for: Automatic Identification of Systolic Time Intervals in Seismocardiogram
Source: Sci Rep. 2016 Nov 22;6:37524. doi: 10.1038/srep37524 (PMC5118745; doi:10.1038/srep37524)

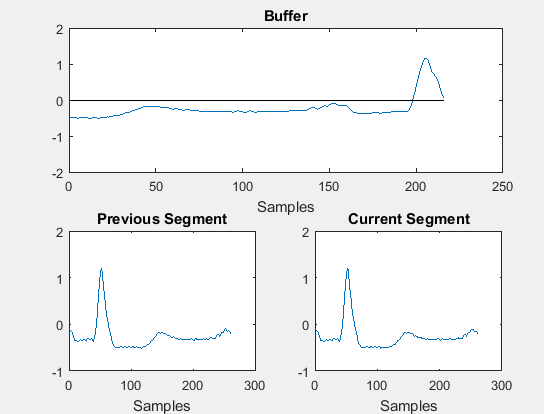

Supplement: Supplementary Video S1 [file srep37524-s2.gif]
